# Force Transfer and Stress Distribution in an Implant-Supported Overdenture Retained with a Hader Bar Attachment: A Finite Element Analysis

**DOI:** 10.1155/2013/369147

**Published:** 2013-12-26

**Authors:** Preeti Satheesh Kumar, Kumar K. S. Satheesh, Jins John, Geetha Patil, Ruchi Patel

**Affiliations:** ^1^Department of Prosthodontics, The Oxford Dental College & Research Hospital, Bommanhalli, Hosur Road, Bangalore 68, India; ^2^Department of Prosthodontics, Rajiv Gandhi College of Dental Sciences, Bangalore, India; ^3^Department of Prosthodontics, Ahmedabad Dental College, India

## Abstract

*Background and Objectives.* A key factor for the long-term function of a dental implant is the manner in which stresses are transferred to the surrounding bone. The effect of adding a stiffener to the tissue side of the Hader bar helps to reduce the transmission of the stresses to the alveolar bone. But the ideal thickness of the stiffener to be attached to the bar is a subject of much debate. This study aims to analyze the force transfer and stress distribution of an implant-supported overdenture with a Hader bar attachment. The stiffener of the bar attachments was varied and the stress distribution to the bone around the implant was studied. *Methods.* A CT scan of edentulous mandible was used and three models with 1, 2, and 3 mm thick stiffeners were created and subjected to loads of emulating the masticatory forces. These different models were analyzed by the Finite Element Software (Ansys, Version 8.0) using von Mises stress analysis. *Results.* The results showed that the maximum stress concentration was seen in the neck of the implant for models A and B. In model C the maximum stress concentration was in the bar attachment making it the model with the best stress distribution, as far as implant failures are concerned. *Conclusion.* The implant with Hader bar attachment with a 3 mm stiffener is the best in terms of stress distribution, where the stress is concentrated at the bar and stiffener regions.

## 1. Introduction

As life spans lengthen, a significant number of people outlive their teeth. Treating older patients, especially those with disabilities, may be a demanding challenge. Lack of retention and stability is one of the major complaints of edentulous patients [1]. The introduction of osseointegrated implants into dentistry has provided new alternatives for the rehabilitation of edentulous patients. Mandibular implant retained overdentures can provide an effective treatment modality for these patients and, in particular, those who have persistent problems with a conventional mandibular prosthesis [2, 3]. Implant-supported overdentures have gained acceptance over the complete denture because of its relative simplicity, increased comfort and chewing efficiency, greater satisfaction, preservation of residual ridge, retention, stability, and improved patient quality of life [1–4].

Retention for the mandibular implant-supported overdentures is commonly achieved by ball attachments, clip on bar connecting the implants, or magnetic attachments [4]. These retentive attachments generate forces and stresses that differ from those seen with natural teeth supported by periodontal ligament. If these stresses exceed the physiological limit they may lead to several undesirable results [3, 5]. Also the long-term function of a dental implant system will depend on the biomechanical interaction between bone and implant [6]. The masticatory forces induce axial forces and bending moments, which could result in stress on the implant as well as the surrounding bone. Bone tissue is known to remodel its structure in response to mechanical stress. Variations in the internal state of stress in the bone determine whether constructive or destructive remodeling will take place. Low stress levels around a dental implant system may result in disuse atrophy similar to the loss of alveolar crest after the removal of the natural tooth. On the other hand abnormally high stress concentrations in the supporting tissues can result in pressure necrosis and subsequently in the failure of the implant. Studies have demonstrated that implants retaining overdentures are subject to both axial and transverse forces, the latter being smaller but potentially more harmful [7, 8]. Thus it is desirable to study stress distribution through the prosthesis and implants to the supporting bone.

Considering bar attachment implants and the stress distribution around them, it was seen that when a bar fixed to two implants is used, different shapes provide different results. A Hader bar, which is round in cross section, may be preferred over a rigid oval bar attachment system [3] as it helps in better stress distribution around the implants. But it is not clearly known whether it is the rigid implant body or the cervical region of the implant or the Hader bar that is subjected to the most stress concentration and hence subject to fracture and subsequent failure. The effect of adding a stiffener to the tissue side of the Hader bar reduces the transmission of the stresses to the alveolar bone. But the ideal thickness of the stiffener to be attached to the bar is a subject of much debate. Also limited literature exists as to the exact mechanism of force transfer and stress distribution along the Hader bar and the implants. Hence this study aims to analyse the force transfer and stress distribution of an implant-supported overdenture with a Hader bar attachment. The stiffener of the bar attachments was varied and the stress distribution to the bone around the implant was studied.

Methods for the evaluation of stress around dental implant systems include mechanical stress analysis, Photo elasticity, and strain measurement on bone surfaces. These techniques have certain limitations such as difficulties in modifications after modeling. In the past two decades, finite element analysis has become an increasingly useful tool for predicting the effects of stress on implant and surrounding bone thus making it an effective computational tool that has been adapted from the engineering arena to dental implant biomechanics [8]. Hence the finite element analysis was used to analyse the force transfer and stress distribution of an implant-supported overdenture with a hader bar attachment.

## 2. Materials and Method

### 2.1. Construction of Geometric Model

#### 2.1.1. Modeling of the Bone

**Figure 1:**
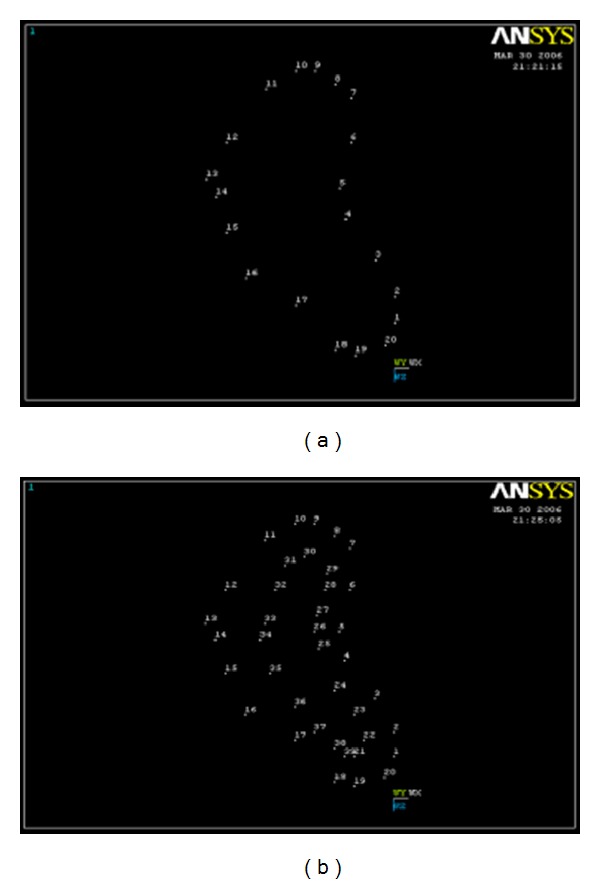
Transformation of profiles into *x*, *y*, and *z* points.

**Figure 2:**
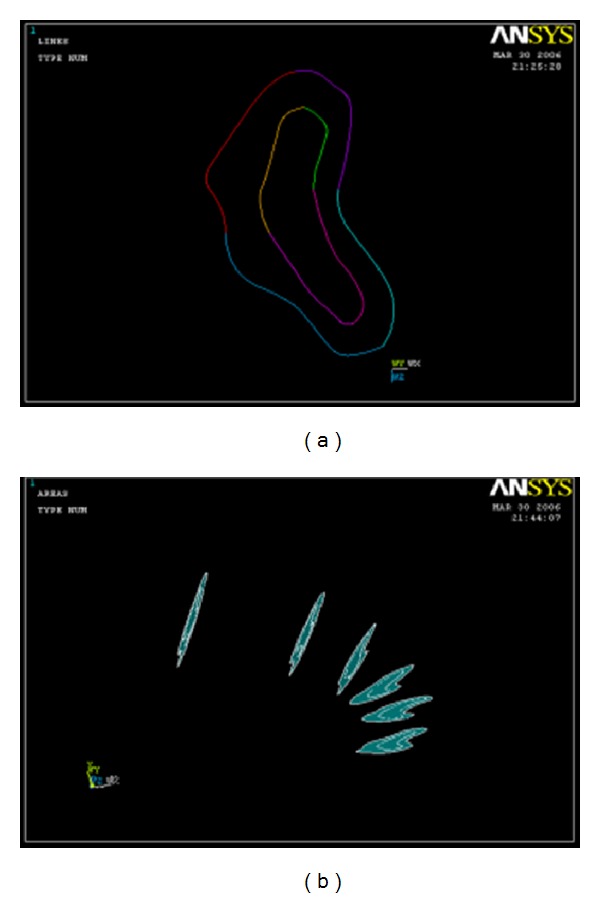
Line geometry and surface geometry of cortical bone.

The algorithm in this study was to generate finite element models from computerized tomography scan (CT scan) data, which was based on the study [5, 9, 10], wherein a CT scan of human mandible was taken and each section from middle to mental foramen was projected on a graph paper. The bone contour and the border between cortical and cancellous bone were traced. The contour data of the profiles were transformed into the *x*, *y*, and *z* coordinate points and read by Finite element program (Figure 1). Connecting these coordinate points gave line geometry also called wire frame modeling. Connecting the lines of each section gave surface geometry also called surface modeling (Figure 2).

**Figure 3:**
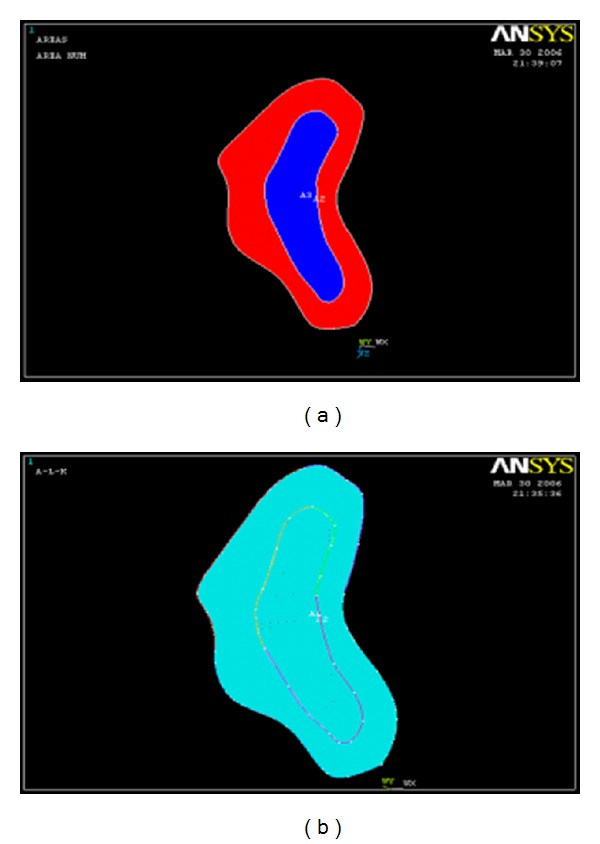
Solid geometry of cortical and cancellous bone.

**Figure 4:**
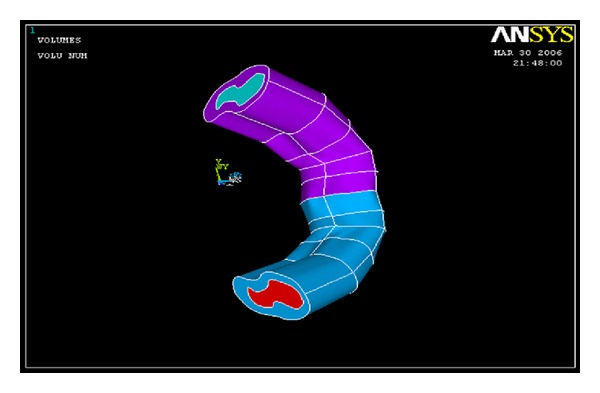
Anatomical model of mandible after superimposition of the cortical and cancellous bone.

Three-dimensional volumes were created from connected successive profiles to define the final solid geometry of cortical bone. The modeling of the cancellous bone was done separately in the same way to get the solid geometry (Figure 3). The final anatomical model was obtained by superimposing both models over each other. This sequence done on one side was repeated to obtain the opposite side (Figure 4).

Through this process, the CT scan data was converted into Three-dimensional solid model of the interforaminal region of edentulous mandible [11].

#### 2.1.2. Modeling of Implant and Superstructure

The titanium-aluminium-vanadium (Ti 6A1-4V) implant used in this study was of a tapered truncated cone design, 12 mm in length and 3.75 mm in diameter with 5° taper [12]. The implant body is covered with a porous coating, titanium plasma spray [13]. The ideal distance between the implants is in the 20–22 mm range [13]. Two solitary implants were placed at 10 mm distance from the midline. The bar attachment has a round superior aspect and an apron below. The apron acts as a stiffener to improve the strength of the bar and limit its flexibility [13]. For this study three thicknesses of the stiffener were modeled leading to three-different models: model A with a 1 mm stiffener, model B with 2 mm stiffener, and model C with 3 mm stiffener.

#### 2.1.3. Modeling of Interface

The implants were designed with a solid machined core of tapered truncated cone and a porous coating [14, 15] consisting of two to three layers of micro spheres. These spherical particles have an average diameter of 100 *μ*m and a porous coating of 300 *μ*m thickness [12, 16]. The powder particle was chosen so that the resulting pore size would be amendable to bone tissue ingrowth [17]. Since this geometry was impossible to implement in light of the grid size used in the model, an analogous set of interface properties was developed. A row of thin interface elements was placed between the porous root and the bone to provide a means of modeling the interface region associated with tissue ingrowth. According to studies [14] done by Cook et al. [18, 19], it was assumed that the bone could be approximated by small cantilever beams in the porous section of the implant at the interface. To relate this to the finite element model, the interface element was assumed to be rectangular cantilever beam of uniform dimension [18].

#### 2.1.4. Modeling of Mucosa

The mucosa was modeled over the cortical bone of uniform thickness of 2 mm on the simplified 3D model. The mucosa was not incorporated in the final anatomical 3D model as it was not significant from the stress analysis point of view and its modulus of elasticity is 1 Mpa that is several orders less than that of the surrounding structures (Implant—110000 Mpa, cancellous bone—9500 Mpa, and cortical bone—26600 Mpa) [7].

#### 2.1.5. Modeling of Overdenture (Acrylic Resin)

The mandibular overdenture was designed to fit the model of the implant and its superstructures. This could not be incorporated in the final anatomical Three-dimensional model due to limitations of the Ansys level 8.0 version.

### 2.2. Preparing of Finite Element Mesh

**Figure 5:**
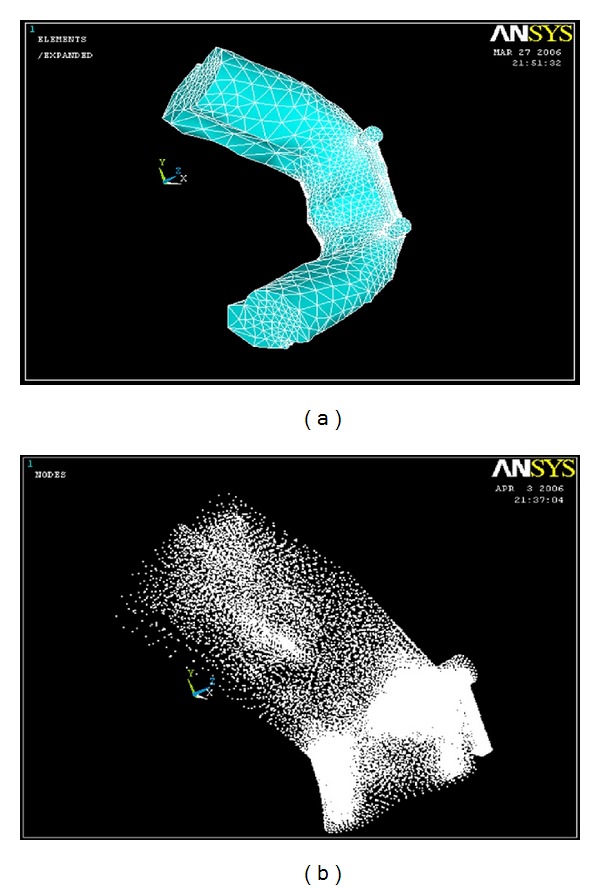
Finite element plot of the completed model and nodal plot for the completed model.

The Three-dimensional finite element model corresponding to the geometric model was generated using ANSYS's Pre-Processor. Care was taken during meshing to concentrate elements in the region of greatest interest of stress distribution pattern. Therefore, Default element size with SOLID 187 element was selected. It is a higher order three-dimensional 10-node element with quadratic displacement behaviour, which is well suited for modeling irregular meshes (such as those produced from various CAD/CAM Systems). The element was defined as 10 nodes having three degrees of freedom at each node: translations in the nodal *x*, *y* and *z* directions (Table 1). The elements were constructed so that their size aspect ratio would yield reasonable solution accuracy. The completed anatomical model consisted of a total number of 45061 nodes and 63193 elements with 1,35,183 degrees of freedom (Table 2 and Figure 5).

### 2.3. Material Properties

All the vital tissues (cortical bone, cancellous bone, and mucosa), implant with superstructure, and acrylic resin were presumed to be linearly elastic, homogenous, and isotropic [16, 21, 22]. Although cortical bone has anisotropy [21] material characteristics and possesses regional stiffness variation, they were modeled isotropically [19] due to the unavailability of sufficient data and difficulty in establishing the principal axis of anisotropy. The corresponding elastic properties such as Young's modulus © and Poisson's ratio (*δ*) of cortical bone, implant, and the bar attachment with stiffener were determined according to literature survey [7, 20].

The model was assigned material properties shown in Table 3.

### 2.4. Application of Boundary Conditions

**Figure 6:**
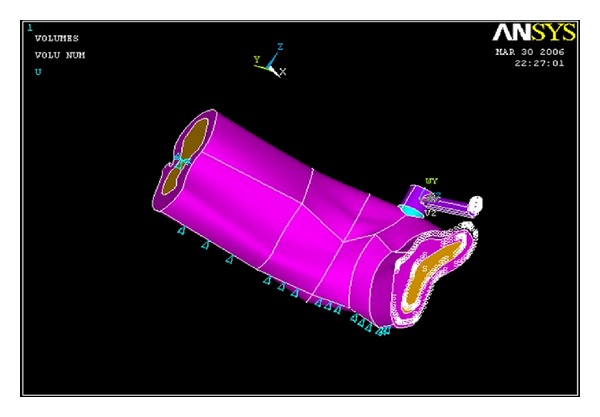
Setting the boundary conditions (blue triangles).

For the boundary condition of the model, a supporting system was set up. Symmetrical boundary conditions were imposed at the mid symphyseal region [8]. On the distal side all the three translations were fixed indicated by light blue color triangles (Figure 6).

### 2.5. Application of Different Loads

The magnitude and the direction of the loading forces were derived from the studies [23–25]. The loads applied were 35 N vertical load applied at 90° to the abutment in a occlusogingival direction [6], 10 N horizontal load applied at 0° over the abutment in a labiolingual direction [6], and 70 N oblique load applied at 120° to the occlusal plane on the abutment in a labiolingual direction, simulating the load from the muscles of mastication [6].

### 2.6. Analysis of Stress Pattern

Three models A, B, and C, each with 1, 2, and 3 mm thick stiffeners, respectively, were used for the load application and analysis. A vertical (35 N), horizontal (10 N) and oblique (70 N), emulating the masticatory load, periodontal force and the muscle force respectively were in turn applied to each of the above models.

These different models were analyzed by the Processor and displayed by *PostProcessor of the Finite Element Software *using von Mises stress analysis. von Mises stress values are defined as the beginning of deformation for ductile materials such as metallic implants. Failure occurs when von Mises stress values exceed the yield strength of an implant material. Therefore they are important for interpreting the stresses occurring within the implant material.

## 3. Results

The stress analysis executed by ANSYS provided results that enabled the tracing of von Mises stress field in the form of color-coded bands. The stress distribution was represented with different color-coding. Red being the highest was followed by orange, yellow light green, green, light blue, blue, and dark blue colors representing the stresses in the descending order. With these different colors the stress distribution pattern can be analyzed in the different models. The corresponding stress values for that particular color are also given at the bottom end of the photographs.

### 3.1. Distribution of the Stresses in the Implant for the Horizontal Load of 10 N (Table 4)

**Figure 7:**
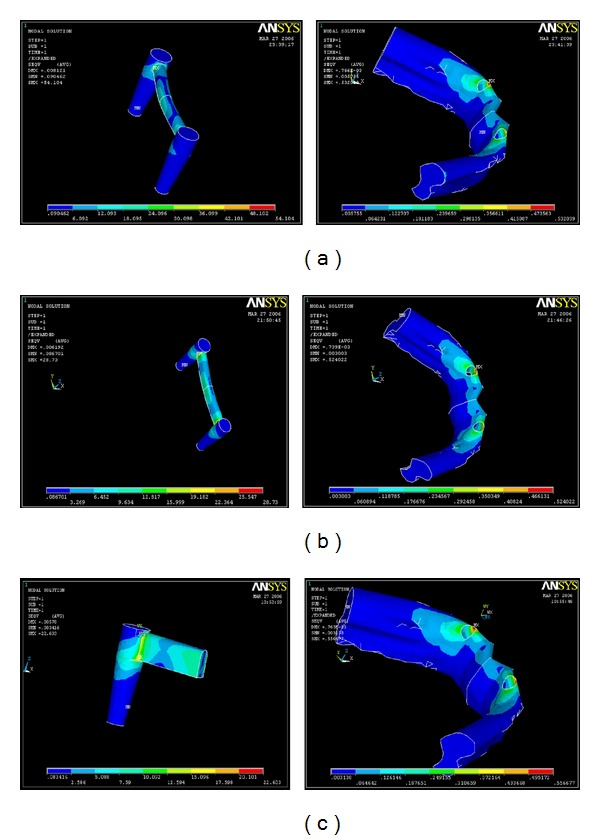
(a) Stress distribution in various parts of the implant and bone with a 1 mm stiffener on a horizontal load of 10 N. (b) Stress distribution in various parts of the implant and bone with a 2 mm stiffener on a horizontal load of 10 N. (c) Stress distribution in various parts of the implant with a 3 mm stiffener with a horizontal load of 10 N.

Under the horizontal load application of 10 N, the maximum stress was found to be congregated at the Cervical region for Models A and B, the values being, 6.092 MPa and 9.634 MPa respectively. Only in Model C, 3 mm stiffener, the maximum stress concentration was in the body of the implant, 10.092 MPa. The least stress was seen in the Abutment for all three models, the values being 0.091 MPa, 0.087 MPa and 0.083 MPa for models A, B and C, respectively (Table 4) (Figures 7(a), 7(b), and 7(c)).

### 3.2. Distribution of the Stresses in the Implant for the Vertical Load of 35 N (Table 5)

**Figure 8:**
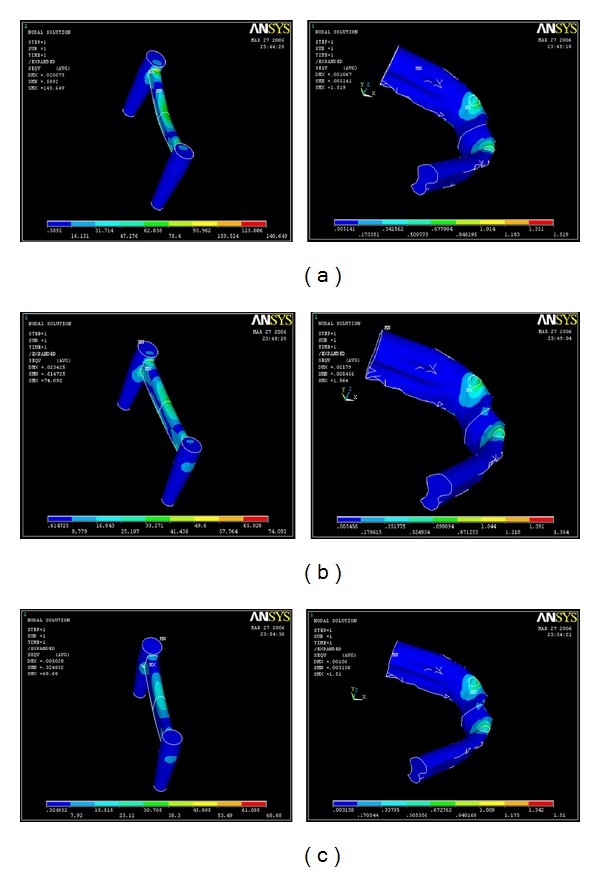
(a) Stress distribution in various parts of the implant and bone with a 1 mm stiffener for a vertical load of 35 N. (b) Stress distribution in various parts of the implant and bone with a 2 mm stiffener for a vertical load of 35 N. (c) Stress distribution in various parts of the implant and bone with a 3 mm stiffener for a vertical load of 35 N.

Under the vertical load application of 35 N, the maximum stress was found to be concentrated at the Hader bar for all three models, the values being, 62.838 MPa, 33.271 MPa, and 15.515 MPa for models A, B and C respectively. The least stress was seen in the abutment and body of the implant for all three models, the values being, 0.590 MPa, 0.615 MPa, and 0.325 MPa for models A, B, and C, respectively (Table 5) (Figures 8(a), 8(b), and 8(c)).

### 3.3. Distribution of the Stresses in the Implant for the Oblique Load of 70 N (Table 6)

**Figure 9:**
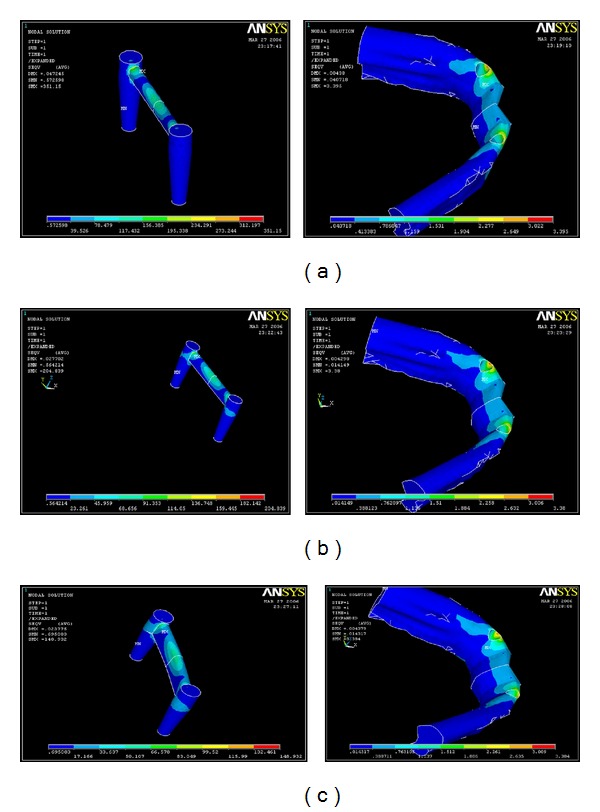
(a) Stress distribution in various parts of the implant and bone with a 1 mm stiffener for an oblique load of 70 N. (b) Stress distribution in various parts of the implant and bone with a 2 mm stiffener for an oblique load of 70 N. (c) Stress distribution in various parts of the implant and bone with a 3 mm stiffener for an oblique load of 70 N.

Under the oblique load application of 70 N, the maximum stress was found to be concentrated at the Hader bar for all three models, the values being 156.385 MPa, 45.959 MPa and 50.107 MPa followed by the cervical region (78.479, 23.261, and 33.637 MPa) for models A, B, and C, respectively. The least stress was seen in the Abutment and the implant body for all three models, the values being 0.573 MPa, 0.564 MPa, and 0.695 MPa for models A, B, and C respectively (Table 6) (Figures 9(a), 9(b), and 9(c)).

**Figure 10:**
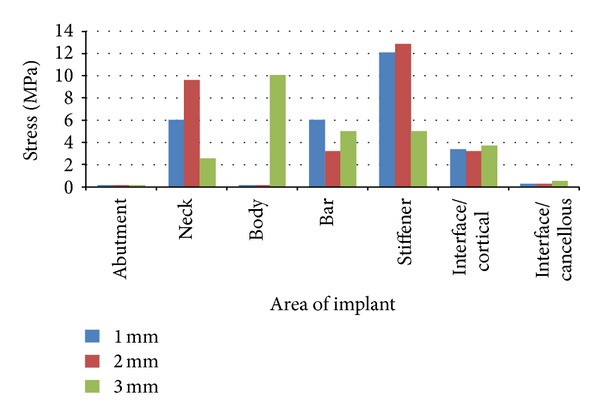
Comparison of stress distribution in various parts of the implant under a horizontal (10 N) load.

**Figure 11:**
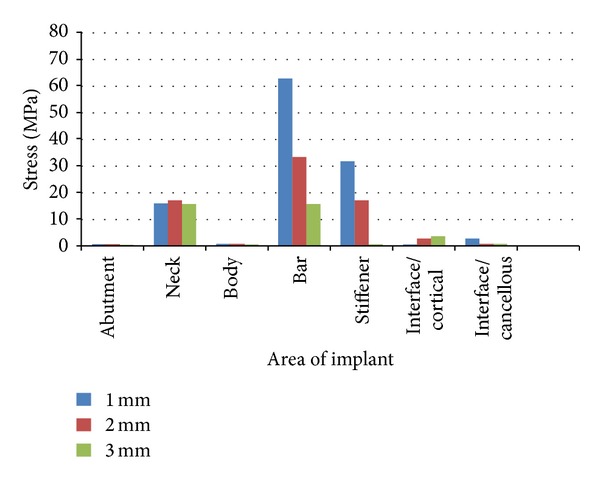
Comparison of stress distribution in various parts of the implant under a vertical (35 N) load.

**Figure 12:**
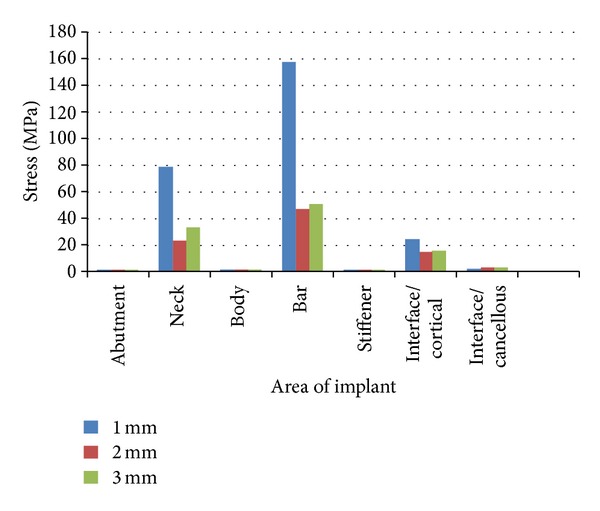
Comparison of stress distribution in various parts of the implant under an oblique (70 N) load.

For a better understanding the above results were formulated into tabular columns and a bar graph was plotted to study the stress patterns generated in the cortical and trabecular bone with the different models at different loadings (Figures 10, 11, and 12).

## 4. Discussion

Implant dentistry has helped greatly in improving the treatment options that are available for the edentulous patient. The ability to replace lost teeth with osseointegrated implants has improved the quality of life especially for edentulous patients [2]. The primary reason for restoration of the edentulous mandible with implant-stabilized prosthesis is the improved function and comfort associated with minimizing or eliminating movement of the mandibular overdenture. Such an implant-supported overdenture, using the two implant treatment modality, is subjected to various types of axial and non-axial stresses, including the masticatory forces. The result of these forces is, obviously, resorption of the surrounding alveolar bone and more importantly concentration of stresses in the different parts of the implants, leading to implant failure. In fact, improper loading of an implant has been demonstrated to be the most common cause of failure of implant therapy.

This has led to many innovations in the implant designs, which attempt to minimize these detrimental effects of the normal masticatory loads, and hence function to increase the longevity of the implant-supported overdenture. Among these modernizations is the use of a stress-breaker-like attachment between the two implants, like a Hader bar (instead of rigid bar, like the Dolder bar), with connecting clips which help in better distribution of the stresses around the implants. But it is not clearly known whether it is the rigid implant body or the cervical region of the implant or the Hader bar that is subjected to the most stress concentration and hence subject to fracture and subsequent failure. The effect of adding a stiffener to the tissue side of the Hader bar has been to reduce the transmission of the stresses to the alveolar bone. But the ideal thickness of the stiffener to be attached to the bar is a subject of much debate.

A dodging problem that has been seen with using osseointegrated dental implants are the amount of stress that is transferred from the implant to the surrounding bone. Studies [3] have shown that clear differences exist in the way stresses are transferred to the bone in a tooth-supported overdenture and an implant-supported overdenture. The main difference cited was the absence of relative movement in response to load transfer from root analog to bone in osseointegrated implants. Titanium implants are stiffer than natural teeth and tend to transmit and distribute greater stresses to adjacent bone. Excessive stresses developed at bone-implant interface could cause the degradation of osseointegration and the failure of the treatment.

Hence there is a need to know and properly understand the biomechanics of the stress transfer from the prosthesis to the implant unit (attachment, implant, and surrounding bone) in two-implant-supported overdenture.

This study is for the most part directed to determining the stress patterns generated around implants with bar attachments. The factors which influence the distribution of stresses around the implant-supported overdenture and the alveolar bone include the implant design, surface topography, type of bar attachment, the type of load, and the thickness of the stiffener among others. This study also undertakes to explore the effect of these factors on the stress distribution and endeavors to rationalize the cause-effect relationship between them.

In a two-dimensional method it is not possible to study horizontal or oblique bite forces. Therefore it is not a valid representation of a clinical situation [8]. To suit the aims of this study, a Three-dimensional finite element model was generated, which is well suited to study the true biomechanical behavior in localized regions of major supporting hard tissues of the mandible. Certain assumptions were made in geometric considerations, material properties, boundary conditions and bone implant interface to make modeling and solving process possible [26, 27]. It is apparent that the presented model was only an approximation of the clinical situation therefore, it is advisable to focus on qualitative comparison rather than quantitative data from these analyses.

## 5. Model Considerations

A mechanical model of an edentulous mandible was generated from computerized tomography (CT scan) as it can give exact bony contours of cancellous and cortical bones [27, 28].

Some parameters were not considered in this study; with complexity of the geometry during meshing the number of elements may exceed the operating capacity of the software and hence may require sub structuring or other alternatives to conduct an analysis. Therefore these parameters were evaluated on a comparatively simple Three-dimensional model. The stress found on mucosa and overdenture was not found to be significant and neither did it affect the purpose of this study. The ramus and condyles of the mandible were also not modeled to save the modeling time, computer memory, processing time, and ease for analysis. To simulate muscle forces, the boundary conditions were applied at the distal end of the mandible. Meijer et al. [6] reported in a Three-dimensional study that similar results were obtained when the entire mandible was modeled with loading and boundary conditions approximating the physiologic ones and with simulation of only interforaminal region.

The accuracy of the results decreases with the increase in elements size. However, for this study, the gradual increase in element size protected the area of interest from being affected by the inaccuracies of the stresses in large elements. The acceptable percentage of error for FEA model should be less than 3% and here it is 0.3%. The results of this analysis concur with the findings of other studies that have used different investigation methods. Therefore the model employed in this study is considered to satisfactorily simulate reality.

## 6. Material Properties

Material properties and their structural basis help us to understand the bone quality type. Material properties greatly influence the stress and strain distribution in the structure. All the vital tissues (cortical bone, cancellous bone and mucosa) implant with superstructure and acrylic resin were presumed to be linearly elastic, homogenous, and isotropic [16, 21, 22].

## 7. Type of Loading

The magnitude of the bite force is dependent on the force direction. In the present study three forces from different directions were selected: a horizontal bite force, a vertical bite force, and an oblique bite force. The proportion of the force magnitude was 1 : 3.5 : 7, respectively [8, 24].

The vertical bite force was determined to be 35 N from studies which measured the bite force of edentulous patients with overdentures supported by implants in the mandible [25, 29]. This value was substituted in the above equation to derive the forces in the other directions. The loading force for the horizontal direction is 10 N, for the vertical direction it is 35 N, and for the oblique direction it is 70 N. The horizontal force is applied in the lingual direction to simulate the constant force applied by the tongue. The oblique force is applied on the buccal surface to simulate the chewing forces.

## 8. Stress Distribution

The von Mises stresses are most commonly reported in finite element analysis studies to summarize the overall stress at a point. Both compressive and tensile stresses, if in excess, can lead to bone resorption and necrosis. Thus, the factors that may lower the overall amount of potentially harmful stress to the bone were investigated by comparing the equivalent stress in models.

## 9. Implants

The titanium-aluminium-vanadium (Ti 6A1-4V) implants used in this study was of a tapered truncated cone design, 12 mm in length and 3.75 mm in diameter with 5° taper [30]. Rieger concluded that a cylindrical implant design directed most of the applied axial load to the apical bone while the tapered design provided better stress distribution [12]. Therefore a tapered design was designed for this study. The implant body is covered with a porous coating, titanium plasma spray [13]. The ideal distance between the implants is in the 20–22 mm range [13]. Two solitary implants were placed at 10 mm distance from the midline. The bar attachment, has a round superior aspect and an apron below. The apron acts as a stiffener to improve the strength of the bar and limit its flexibility [13].

The findings of the present study are discussed under the following headings.

### 9.1. Distribution of the Stresses in the Implant for the Horizontal Load of 10 N

Under the horizontal load application of 10 N, the maximum stress was found to be congregated at the neck of the implant for Models A and B. Only in Model C, 3 mm stiffener, the maximum stress concentration was in the body of the implant. The least stress was seen in the abutment for all three models (Figures 7(a), 7(b), and 7(c)).

### 9.2. Distribution of the Stresses in the Implant for the Vertical Load of 35 N

Under the vertical load application of 35 N, the maximum stress was found to be concentrated at the Hader Bar for all three models. The least stress was seen in the abutment and Body of the implant for all three models (Figures 8(a), 8(b), and 8(c)).

### 9.3. Distribution of the Stresses in the Implant for the Oblique Load of 70 N

Under the oblique load application of 70 N, the maximum stress was found to be concentrated at the Hader Bar for all three models, followed by the neck of the implant for models A, B, and C, respectively. The least stress was seen in the Abutment and the implant body for all three models. (Figures 9(a), 9(b), and 9(c)).

This is in accordance with the study by Papavasiliou et al. [31] and Yokoyama et al. [32], where a consistent observation from all models was concentration of maximum stress at the bone-implant interface at the level of cortical bone. It also concurs with Meijer et al. [6, 8] who reported that the most extreme stress values in all models were located around the neck of the implant. This might be explained as a result of the stress transferring mechanism that occurs in the implant bone complex [32]. High stresses transmitted through the implant concentrate at the level of the cortical bone along the facial surface of the implant. The stresses decrease on encountering cancellous bone [33].

### 9.4. Stress Distribution in the Implant Abutment under Loading Conditions

The maximum stress was found concentrated in models B and C, the ones with 2 mm and 3 mm stiffener, under the horizontal and oblique loading, respectively, whereas when a vertical load of 35 N was applied the model A, with 1 mm thick stiffener showed the maximum stress. The least stress concentration around the abutment of the implant was in model C, in model B, and in model A, all for the horizontal load in ascending order.

### 9.5. Stress Distribution in the Cervical Region of the Implant under Loading Conditions

The maximum stress was found concentrated in model A, model B, and model C, the one with 1 mm and 3 mm stiffener, under the oblique loading. The least stress concentration around the neck of the implant was in model C, model A, and in model B, respectively, in ascending order for the horizontal load.

### 9.6. Stress Distribution in the Implant Body under Loading Conditions

The maximum stress was found concentrated in Model C, the one with 3 mm stiffener, under the horizontal and oblique loading, respectively, whereas when a vertical load of 35 N was applied the Model B, with 2 mm thick stiffener showed the maximum stress. The least stress concentration around the body of the implant was in model B and model A, for the horizontal load. In model C, the least stress concentration in the body was seen for the vertical load.

### 9.7. Stress Distribution in the Hader Bar under Loading Conditions

The maximum stress was found concentrated in model A, the one with 1 mm stiffener, under the oblique and vertical loading. The maximum stress concentration in models C and B were seen for the Oblique loading. The least stress concentration around the Hader bar was in models B, C, and A, in that order, for the horizontal load.

### 9.8. Stress Distribution at the Cortical Bone Interface under Loading

The maximum stress was found concentrated in model A, the one with 1 mm stiffener, under the oblique loading. The maximum stress concentration in models B and C were seen for the Oblique loading respectively. The least stress concentration around the Hader bar was in models A, B, and C for the vertical load in ascending order.

### 9.9. Stress Distribution at the Cancellous Bone Interface under Loading Conditions

The maximum stress was found concentrated in model C, the one with 3 mm stiffener, under oblique loading. The maximum stress concentration in models A and B were seen for the vertical and oblique loading, respectively. The least stress concentration around the Hader bar was in models B, A, and C, for the horizontal load in ascending order.

Stresses induced by occlusal loads are initially transferred from the implant to the cortical bone, while a small amount of remaining stress is spread to the cancellous bone. It is also possible that higher strain values are observed in cortical bone as it presents a higher elastic modulus when compared with trabecular bone [1, 6, 16, 32] and thus has a greater ability to transfer stress [32]. These findings are in accordance with other in vitro studies [6, 7, 28].

This finite element study demonstrated that axial loading shows the least stress, better stress homogenization and gives a favorable prognosis for an implant. Therefore the forces should be directed along the long axis of the tooth. This can be achieved by carefully planning the occlusion and type of superstructure to be used.

Nonaxial loading has been related to marginal bone loss, failure of osseointegration, and failure of implant and/or prosthetic superstructure component. It is clear that during normal chewing the highest stress occurred due to oblique bite forces. To reduce high stress levels efforts must be made to reduce large bite forces from oblique directions. The direction of the bite force cannot be changed in patients, but the magnitude can be influenced by the design of the prosthesis.

Understanding of the forces and patterns of stress distribution in the bone underneath the denture is a major factor during the planning of denture fabrication. Neglect of these factors may result in unnecessary discomfort to the denture wearer and cause alveolar ridge resorption [34].

## 10. Limitations of Finite Element Modeling

The present study has certain limitations; firstly the vital anisotropic tissues were considered isotropic. Next the loads applied were static loads that were different from dynamic loading seen during function. This study has limitations when predicting the response of biological systems to applied loads, as do all modeling systems, including photoelastic analysis and strain gauge measurements. Hence even though finite element analysis provides a sound theoretical basis of understanding the behavior of a structure in a given environment, it should not be considered alone. Actual experimental techniques and clinical trials should follow finite element analysis to establish the influence of observed stress levels on the tissue and prosthesis function.

## 11. Conclusion

Within the limitations of the study, the following conclusion can be drawn.

1. The region which displayed the maximum stress concentration in the models A and B was the neck of the implant, when compared to the bar attachment of the same models.
2. In model C the maximum stress concentration was in the bar attachment making it the model with the best stress distribution, as far as implant failures are concerned.
3. The stress in the implant decreased as the thickness of the stiffener in the bar attachment increased.
4. Regarding supporting tissues, the maximum stress values were concentrated in the cortical bone and were observed mainly around the neck of the implant.

## Figures and Tables

**Table 1:** Distance vector components used for the finite element modeling.

*x* direction	*y* direction	*z* direction
0	28.07	33.01
0	30.61	5.27
0	9.56	6.31
0	27.67	38.97
0	80.63	23.89

**Table 2:** Mesh data—number of elements, nodes, and degrees of freedom.

Region	Elements	Nodes	Degrees of Freedom
Implant	10418	15868	31.254
Interface	3977	8263	11,931
Outer (cortical) bone	15707	25248	47,121
Inner (cancellous) bone	14964	25739	44,892
Hader bar	1352	1996	4,506
Complete model	45061	63193	1,35,183

**Table 3:** Material properties [7, 20] assigned to the model.

	Young's modulus (MPa)	Poison's ratio
Implant	103400	0.35
Interface	54450	0.325
Cancellous bone	5500	0.3
Cortical bone	28500	0.3

**Table 4:** Comparison of the stress distribution in the various parts of the implant under a horizontal (10 N) load.

	Abutment	Cervical region	Body	Bar	Stiffener	Interface/cortical	Interface/cancellous
Model A 1 mm	0.090	6.092	0.090	6.092	12.093	3.403	0.236
Model B 2 mm	0.087	9.634	0.087	3.269	12.817	3.285	0.235
Model C 3 mm	0.083	2.586	10.092	5.088	5.088	3.735	0.495

**Table 5:** Comparison of the stress distribution in the various parts of the implant under a vertical (35 N) load.

	Abutment	Neck	Body	Bar	Stiffener	Interface/cortical	Interface/cancellous
Model A 1 mm	0.590	16.151	0.590	62.838	31.714	0.510	2.73
Model B 2 mm	0.615	16.943	0.615	33.271	16.943	2.725	0.698
Model C 3 mm	0.325	15.515	0.325	15.515	0.325	3.466	0.673

**Table 6:** Comparison of the stress distribution in the various parts of the implant under an oblique (70 N) load.

	Abutment	Neck	Body	Bar	Stiffener	Interface/cortical	Interface/cancellous
Model A 1 mm	0.573	78.479	0.573	156.385	0.573	23.304	1.904
Model B 2 mm	0.564	23.261	0.564	45.959	0.564	14.373	3.006
Model C 3 mm	0.695	33.637	0.695	50.107	0.695	14.6	3.009
